# The Release of Bound Phenolics to Enhance the Antioxidant Activity of Cornmeal by Liquid Fermentation with *Bacillus subtilis*

**DOI:** 10.3390/foods14030499

**Published:** 2025-02-04

**Authors:** Ping Zhang, Jialan Zhang, Li Li, Tong Gu, Suo Chen, Jinsong Wang, Mengxiang Gao

**Affiliations:** 1 College of Life Science, Yangtze University, Jingzhou 434025, China; 2022721035@yangtzeu.edu.cn (P.Z.); lily2012@yangtzeu.edu.cn (L.L.); gutong1117@126.com (T.G.); chensuo9803@126.com (S.C.); 2 College of Animal Science and Technology, Yangtze University, Jingzhou 434025, China; 3 Institute of Food Science and Technology, Yangtze University, Jingzhou 434025, China; 4 College of Bioengineering, Jingchu University of Technology, Jingmen 448000, China; jswang@jcut.edu.cn

**Keywords:** cornmeal, bound phenolics, carbohydrate-hydrolyzing enzymes, antioxidant activity, fermentation, *Bacillus subtilis*

## Abstract

This study investigated the influence of *Bacillus subtilis* fermentation on the composition of phenolic substances and antioxidant activity in cornmeal. The results indicate that the fermentation process significantly increased both the total phenolic content (TPC) and total flavonoid content (TFC). After 5 days of fermentation, the TPC rose from 31.68 ± 1.72 mg/g to 39.46 ± 2.95 mg/g, representing a 24.56% increase, while the TFC increased from 2.13 ± 0.11 mg/g to 7.56 ± 0.29 mg/g, marking a 254.93% increase. Additionally, the proportion of free phenolic compounds in cornmeal increased from 20.24% to 83.98%, while the proportion of bound phenolic compounds decreased from 79.76% to 16.02%. Furthermore, the hydrolytic enzyme activities of cellulase, *β*-glucosidase, and xylanase were significantly correlated with the free phenolic content (FPC) (*r* > 0.85, *p* < 0.05), indicating their crucial role in releasing free phenolic compounds from cornmeal. Employing scanning electron microscopy, differential scanning calorimetry, X-ray diffraction, and Fourier-transform infrared spectroscopy analyses, we inferred that the enzymes produced by the microorganisms disrupted the cellular structure of cornmeal and weakened the interactions between bound phenolics and the food matrix, thereby facilitating the release of phenolic compounds. This release resulted in an overall increase in the antioxidant activity of the cornmeal. The study provided a novel approach to enhancing the bioavailability of phenolic acids in cornmeal, indicating the potential benefits of fermentation in food processing.

## 1. Introduction

Corn (*Zea mays* L.) is recognized as one of the most widely cultivated cereal crops globally for its rapid maturation, high yield, and robust cold tolerance [1]. Corn serves as a staple food with significant nutritional value, providing essential nutrients such as starch, protein, fat, and dietary fiber. Furthermore, it is abundant in phenolic compounds and other bioactive substances, earning a reputation as a “golden food” [2,3,4]. Research has demonstrated that phenolic compounds present in corn, including phenolic acids, flavonoids, and carotenoids, are the most important natural antioxidants [5]. These bioactive compounds demonstrate substantial antioxidant properties. Therefore, the regular consumption of corn may confer numerous health benefits.

Polyphenols, characterized by one or more phenolic hydroxyl groups, represent a class of secondary metabolites widely found in plants. This class includes a variety of compounds such as phenolic acids, flavans, tannins, lignans, and flavonoids [6]. These compounds are classified into two main categories based on their binding characteristics and biological activity: soluble free polyphenols and insoluble bound polyphenols [7]. In corn, the predominant phenolic compounds are found in insoluble forms, binding to polysaccharides and proteins within the cell wall matrix. This binding renders them challenging to utilize, resulting in the lower overall bioavailability of cereal polyphenols [8]. Therefore, it is crucial to convert the phenolic compounds in corn from the bound form to a free form to enhance their solubility and bioavailability. In response to this issue, researchers have devised various strategies to facilitate the release of phenolics from cereals, including chemical, physical, and enzymatic approaches [7,9]. The application of these methods in the food industry is limited due to their distinct characteristics [10].

Microbial fermentation technology is an effective method for the release and transformation of phenolic compounds found in grains and fruits [11]. This technology leverages the complex enzymes produced by microorganisms, such as cellulases, ligninases, and pectinases, to facilitate the degradation of fibrous components in plant cell walls. It breaks the covalent bonds between polyphenols and other substances, thereby enabling the release and conversion of phenolic substances, which may potentially alter their bioactivity [12,13]. For instance, co-fermentation with *Monascus anka* and *Bacillus* sp. significantly enhances the bioactivity of guava leaves [14], as high-activity carbohydrate-hydrolyzing enzymes cleave the chemical bonds connecting guava polyphenols to the cellulose, hemicellulose, and polysaccharide components in the cell wall, resulting in a greater release of soluble polyphenols. Moreover, the solid-state fermentation of black tea with *B. subtilis* LK-1 enhances both its catechin composition and antioxidant activity [15]. The fermentation of black soybeans with *B. subtilis* effectively enhances their total phenolic and flavonoid content, as well as the antioxidant activity of black bean extracts [16]. Solid-state fermentation technology, particularly that driven by fungi, has been extensively applied to the extraction of plant-bound phenols. In contrast, research on bacterial liquid-state fermentation in this field remains relatively limited. However, liquid-state fermentation offers several advantages over solid-state fermentation, such as more efficient mass transfer, higher enzyme activity, greater product homogeneity, and shorter fermentation cycles. Considering the abundance of bound phenols in corn kernels and the established ability of *Bacillus* species to produce hydrolytic enzymes, further investigation into bacterial fermentation is warranted.

This study aims to investigate the effects of liquid-state fermentation with *Bacillus subtilis* on the phenolic compounds and antioxidant activity in corn. Specifically, we will characterize the dynamic changes in total polyphenols, total flavonoids, free phenols, bound phenols, and antioxidant activity throughout the fermentation process. To achieve a comprehensive understanding of the transformations occurring, we will employ structural characterization techniques, such as scanning electron microscopy (SEM), differential scanning calorimetry (DSC), X-ray diffraction (XRD), and Fourier-transform infrared spectroscopy (FT-IR), to compare the structural characteristics of fermented and unfermented cornmeal. Furthermore, we will monitor the dynamic changes in relevant hydrolytic enzymes throughout the fermentation process to elucidate the underlying mechanisms driving phenolic alterations. This study aims to enhance the bioavailability of corn polyphenols and provide innovative strategies for the development high-value functional corn products.

## 2. Materials and Methods

### 2.1. Liquid Fermentation of Cornmeal and Sample Preparation

The kernels of Huawan 617 corn (*Zea mays* L.) purchased from the market were air-dried, ground, and passed through a 20-mesh sieve to produce cornmeal. Ten grams of cornmeal were fully submerged in 100 mL of distilled water, subsequently placed in a 250 mL conical flask, and subjected to high-temperature and high-pressure sterilization prior to use. The *B. subtilis* strain 4-3, obtained from the College of Life Sciences, Yangtze University, was inoculated into Luria–Bertani (LB) medium and incubated at 37 °C and 150 rpm for 14 h. After incubation, the bacterial cells were adjusted to a concentration of 10^8^ CFU/mL by discarding the supernatant through centrifugation. Both the prepared *B. subtilis* suspension and sterile distilled water were each inoculated into the cornmeal medium at a ratio of 4% (*v/v*). The mixture was then cultured at 37 °C and 180 rpm for 7 d. The *B. subtilis*-fermented cornmeal was referred to as fermented cornmeal (FC), while the unfermented cornmeal served as the control, labeled unfermented cornmeal (UC). Samples were collected for analysis at 0, 1, 2, 3, 4, 5, 6, and 7 d of fermentation. The pH of the fermented cornmeal was measured directly using a pH meter (FE28, Mettler Toledo, Shanghai, China). Then, the fermented cornmeal was freeze-dried for 48 h, followed by grinding and passing through a 20-mesh sieve for further analysis.

### 2.2. Determination of Nutritional and Functional Components

The cornmeal was subjected to the analysis of various nutritional components including moisture, starch, protein, crude fat, crude fiber, ash, soluble sugars, and reducing sugars according to Chinese national standards.

The total acid content (TAC) of the cornmeal was determined by the acid–base indicator titration method outlined in Chinese national standards GB12456-2021 [17]. In brief, 0.5 g of cornmeal was blended with 50 mL of distilled water (free of carbon dioxide) for 3 min, and the mixture was then filtered to obtain the filtrate. The filtrate was titrated with a standard sodium hydroxide solution (NaOH, 0.1 mol/L) to a pH of 8.2. Distilled water free of carbon dioxide served as a control to determine the volume of NaOH consumed in the absence of the sample. Subsequently, the TAC in the cornmeal was calculated based on the sodium hydroxide consumption and expressed in g/kg of cornmeal.

Free phenolic compounds were extracted using the methods described by Bei et al. [18] with slight modification. Cornmeal (1 g) was combined with 80% methanol in a 1:20 (*w/v*) ratio and sonicated at 30 °C for 30 min (240 W power and a frequency of 50 Hz) using an ultrasonic instrument (KQ-600DE, Kunshan Ultrasonic Instrument Co., Ltd., Kunshan, China). The mixture was then centrifuged at 7000× *g* and 4 °C for 10 min to collect the supernatant. This extraction process was repeated three times. The collected supernatants were then concentrated to a final volume of 20 mL using a rotary evaporator (Eyela N-1200B, Tokyo Rikakikai Co., Ltd., Tokyo, Japan) and stored at −20 °C for further analysis.

The bound phenolics were extracted using the method described by Li et al. [19] with slight modifications. After the extraction of free phenolics, 20 mL of hexane was added to the residue remaining to defat the sample. Subsequently, 20 mL of NaOH solution (2 mol/L) was introduced into the mixture, which was then purged with nitrogen, sealed, and agitated for 4 h to facilitate hydrolysis. The pH of the solution was adjusted to 2.5. After centrifugation to collect the supernatant, the solution was separated five times with the same volume of ethyl acetate. The ethyl acetate fractions were combined, concentrated, and dried by rotary evaporation at 45 °C, and then filled to 20 mL with 80% methanol. The final solution was stored at −20 °C for subsequent analysis.

The free and bound phenolic content (FPC and BPC) was measured using the Folin–Ciocalteu reagent, as described by Akbari et al. [20]. In brief, the phenolic extract was mixed with a same volume of Folin–Ciocalteu reagent and allowed to stand in the dark for 8 min. Subsequently, the same volume of 7.5% Na_2_CO_3_ was added to the mixture, which was then mixed thoroughly and left at 25 °C for 2 h. The absorbance was measured at 760 nm using a spectrophotometer (UV2600, Shimadzu Co., Ltd., Kyoto, Japan). The results were expressed in gallic acid equivalents (GAE mg/g DW). The total phenolic content (TPC) was calculated as the sum of FPC and BPC.

The total flavonoid content (TFC) was assessed using the NaNO_2_-Al(NO_3_)_3_-NaOH method, with detailed operational procedures outlined by Liu et al. [21]. TFC was expressed in rutin equivalents (RE mg/g DW), and the standard calibration curve was constructed with rutin.

### 2.3. Determination of Enzyme Activity

Fermented cornmeal was mixed with a citric acid buffer (0.05 mol/L, pH 4.8) in a 1:10 (*m/v*) ratio and agitated at 30 °C and 180 rpm for 2 h. Subsequently, the mixture was centrifuged at 4 °C and 7100× *g* for 30 min, and the supernatant was collected as crude enzyme solution for the determination of enzyme activities [22]. In addition to the experimental samples, an inactivated enzyme solution was employed as a control for each enzyme activity assay.

#### 2.3.1. Carbohydrate Hydrolase Activity Assay

The enzymatic activity of sodium carboxymethyl cellulose (CMCase) was determined using the protocol described by Xie et al. [23]. Briefly, the crude enzyme solution was mixed with three volumes of 1% CMC-Na and incubated at 50 °C for 30 min, then boiled for 5 min to halt the reaction. Subsequently, the glucose concentration in the solution was determined using the 3,5-dinitrosalicylic acid (DNS) method. The method for determining the activity of xylanase was analogous to that described in enzymatic activity of CMCase. However, a key difference was that the substrate used in the reaction was 1% xylan.

The activity of *α*-amylase was determined using the DNS method, with minor adjustments using the methods described by Chen et al. [24]. The experimental procedure followed the steps outlined in the enzymatic activity of CMCase, with the exception that the reaction substrate used was a 0.5% soluble starch solution (*w/v*, 0.05 mol/L citric acid buffer, pH 4.8). The duration of the reaction was set to 10 min.

The *β*-glucosidase activity was assessed using the salicin method as described by Qin et al. [25]. This procedure aligned with the description in enzymatic activity of CMCase, except that a 0.5% salicin solution (*w/v*, 0.05 mol/L citric acid buffer, pH 4.8) was employed.

One unit (U) of CMCase or *α*-amylase activity is defined as the amount of enzyme required to liberate 1 µmol of glucose or maltose per hour under the specified assay conditions. Similarly, one unit of *β*-glucosidase or xylanase activity was defined as the amount of enzyme required to liberate 1 μmol of glucose or xylose per minute under the same assay conditions. These results were quantified and expressed in units per gram of dry weight (U/g DW).

#### 2.3.2. Protease Activity Assay

The protease activity was determined using the method described by Chen et al. [24] with minor adjustments. Initially, crude enzyme solution was mixed with casein solution and incubated at 40 °C for 10 min. The reaction was then terminated by a trichloroacetic acid (C_2_HCl_3_O_2_) solution. After thorough mixing, the mixture was allowed to stand for 10 min to precipitate any remaining casein. The supernatant was collected by centrifugation. Subsequently, the collected supernatant was mixed with sodium carbonate (NaCO_3_) solution and Folin–Ciocalteu reagent. This mixture was incubated at 40 °C for 20 min to develop color. The absorbance of the resulting solution was measured at 660 nm using a plate reader (Enspire, PerkinElmer Business Management Co., Ltd., Shanghai, China). Protease activity was quantified, with one unit (U) defined as the amount of enzyme required to release 1 µg of tyrosine from casein per minute under the stated incubation conditions.

### 2.4. Observation of Corn Flour Microstructure Using Scanning Electron Microscopy (SEM)

The microstructure of cornmeal was observed utilizing a SEM (VEGA3, Tescan Co., Ltd., Shanghai, China). After a freeze-drying process, the cornmeal samples were meticulously placed onto a specimen holder with double-sided conductive adhesive tape. Subsequently, the samples were sprayed with a thin film of gold for 65 s [26]. The morphological characteristics of the samples were observed at varying magnifications (1000×, 2000×, and 5000×).

### 2.5. Differential Scanning Calorimetry (DSC)

The thermal stability of the samples was assessed using a DSC (JY-DSC533, Shanghai Jingyi Chemical Materials Co., Ltd., Shanghai, China). A 10 mg freeze-drying sample was accurately measured and placed into an aluminum pan, with an empty aluminum pan serving as the baseline reference. The measurement was performed under a nitrogen atmosphere with a flow rate of 10 mL/min. The temperature was increased at a rate of 20 °C/min, starting from an initial temperature of 10 °C and increasing up to 400 °C [27]. During the analysis, the DSC traces were recorded, and each measurement was performed in triplicate.

### 2.6. Fourier-Transform Infrared (FT-IR) Spectroscopy Analysis

The freeze-dried corn flour was ground with potassium bromide (KBr) at a ratio of 1:100 (*w/w*) under an infrared lamp. The mixture was then pressed into a mold with a pressure of 15 MPa for 60 s. Subsequently, the samples were analyzed using FT-IR spectrometry (FT-IR-680, Tianjin Tianguang Optical Instrument Co., Ltd., Tianjin Rui’an, China). The scanning parameters were set as follows: a resolution of 4 cm^−1^, a wavenumber range from 4000 to 400 cm^−1^, and a total of 32 scans performed on each sample [28].

### 2.7. X-Ray Diffraction (XRD)

After lyophilization, the samples were analyzed using X-ray diffractometer (SmarLab, Rigaku Co., Ltd., Tokyo, Japan) equipped with a Cu K*α* source (*λ* = 1.5418 nm). The analysis was conducted at a scanning speed of 2° per minute across a 2*θ* range of 10° to 90°. The X-ray generator operated at a voltage of 40 kV and a current of 40 mA [29].

### 2.8. Antioxidant Activity Assay

The antioxidant activity of the cornmeal was assessed using 1,1-diphenyl2-picryl hydrazyl (DPPH) radical scavenging activity, ABTS^+^ radical scavenging activity, and ferric reducing antioxidant power (FRAP) values.

The DPPH radical scavenging activity was evaluated following the methods described by Zheng et al. [30]. A methanol extract of the sample was combined with the same volume of DPPH methanol solution (0.1 mmol/L) and allowed to react in darkness at 25 °C for 30 min. The absorbance of the mixture was measured at 517 nm, denoted as *A_sample_*. A control (*A_control_*) was established by replacing DPPH with an equivalent volume of anhydrous methanol, while a blank (*A_blank_*) was prepared by substituting the sample solution with an equivalent volume of anhydrous methanol. The DPPH radical scavenging rate was calculated using the formula: DPPH radical scavenging rate (%) = [1 − (*A_sample_* − *A_control_*)/*A_blank_*] × 100.

The ABTS^+^ radical scavenging activity was assessed using the method described by Ni et al. [31]. The ABTS^+^ reagent was reacted with a potassium persulfate solution at 25 °C in darkness for 16 h to prepare an ABTS^+^ stock solution. This solution was subsequently diluted with methanol to achieve an absorbance of 0.70 ± 0.02 at 734 nm, resulting in the ABTS^+^ working solution. Equal volumes of the ABTS^+^ working solution and sample methanol extract were combined and reacted in darkness at 25 °C for 10 min. The absorbance was measured at 734 nm immediately following the reaction. The ABTS^+^ radical scavenging rate was calculated using the equation: ABTS^+^ radical scavenging rate (%) = [1 − (*A*_1_ − *A*_2_)/*A*_0_] × 100, where *A_1_*, *A_2_*, and *A_0_* represent the absorbance of the ABTS^+^ solution mixed with the sample, the absorbance of the sample without ABTS^+^ solution, and the absorbance of ABTS^+^ solution without sample, respectively.

The FRAP values of the samples were determined according to the methodology outlined by Tan et al. [32]. Briefly, the methanol extract of the sample was mixed with PBS buffer and potassium ferricyanide solution in a proportional manner. The mixture was then incubated at 50 °C for 20 min before the addition of trichloroacetic acid solution, distilled water, and a FeCl3 solution, respectively, each in proportional amounts. This combined solution was allowed to develop color at 25 °C for 10 min. Finally, the absorbance of the mixture was measured at 700 nm using a spectrophotometer. The FRAP value was calculated by subtracting the absorbance of the sample without potassium ferricyanide (*A_i_*) from that of the sample containing potassium ferricyanide (*A_j_*).

### 2.9. Statistical Analysis

The results were calculated from a minimum of three parallel samples. Pearson correlation analysis was performed using Origin Pro 2021 (Origin Lab, Northampton, MA, USA). A one-way analysis of variance (ANOVA) was performed with SPSS 20.0 (SPSS Inc., Chicago, IL, USA), and significance differences between the samples were determined through the Duncan test (*p* < 0.05).

## 3. Results

### 3.1. Changes in Functional Component Content in Cornmeal

The basic nutritional and functional components of the corn used in this experiment are shown in (Table 1). The corn kernels have the highest content of starch, followed by sugar, moisture, and crude protein. The contents of crude fat, crude fiber, and ash are relatively low. The proportion of bound phenolics is 81.98%.

**Figure 1:**
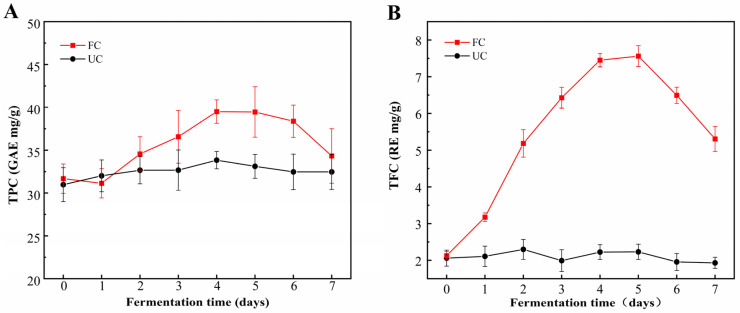

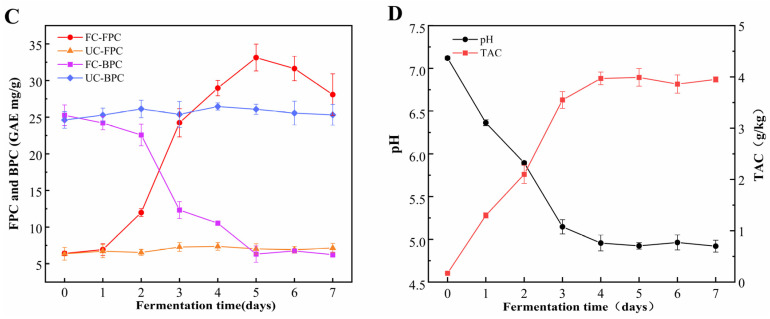
Dynamic changes in various indices during fermentation of cornmeal by *B. subtilis*. (**A**) Total phenolic content (TPC), (**B**) total flavonoid content (TFC), (**C**) free and bound phenolic content (FPC and BPC), (**D**) total acid content (TAC) and pH. Note: UC—unfermented cornmeal, FC—cornmeal fermented with *B. subtilis*.

The fermentation of cornmeal by *B. subtilis* 4-3 significantly enhanced TPC and TFC. This increase in TPC was primarily attributed to elevated levels of free phenols, with a particularly pronounced rise observed on the fifth day of fermentation (Figure 1A–C). After a 5-day fermentation treatment, TPC increased significantly from 31.68 ± 1.72 mg/g to 39.46 ± 2.95 mg/g, reflecting a 24.56% increase. Concurrently, TFC rose from 2.13 ± 0.11 mg/g to 7.56 ± 0.29 mg/g, representing a substantial increase of 254.93%. Moreover, the FPC exhibited a significant increase (*p* < 0.05), rising from 6.41 ± 0.31 mg/g to 33.14 ± 1.84 mg/g, nearly a fivefold increase. In contrast, the BPC decreased from an initial 25.27 ± 1.41 mg/g to 6.32 ± 1.11 mg/g (Figure 1C). Consequently, the proportion of FPC increased to 83.98%, while the proportion of BPC fell to 16.02%. This demonstrates that *B. subtilis* 4-3 possesses the capability to facilitate the conversion of insoluble bound phenols into soluble free phenols during the fermentation of cornmeal. The underlying mechanism may involve the production of various enzymes by microorganisms during the fermentation process, which can hydrolyze large molecular substances in food, thereby disrupting the interactions between polyphenols and the food matrix. This facilitates the transformation of insoluble polyphenols into soluble ones, leading to distinct trends in the levels of free and bound phenols [14]. Additionally, phenolic compounds may be hydrolyzed into smaller molecules during fermentation, leading to an increased exposure of phenolic hydroxyl groups [33]. Moreover, under aerobic conditions, microorganisms can utilize aromatic amino acids such as phenylalanine, tyrosine, and tryptophan to produce new phenolic compounds through aromatic amino acid metabolic pathways [34,35]. In the later stages of fermentation (5–7 d), there is a decline in the contents of TPC and TFC. This decline may be attributed to two main factors: First, as the fermentation progresses, there is a reduction in carbon sources in the cornmeal, prompting microorganisms to utilize certain phenolic compounds as carbon sources for their growth and metabolism [11]. Second, bacteria from the *B. genus* produce polyphenol oxidases, which catalyzes the oxidation of polyphenol compounds into other substances, thereby resulting in a reduction in phenol content [36].

As fermentation progressed, a gradual decrease in pH was observed, accompanied by a significant increase in TAC (Figure 1D). The pH declined from an initial measurement of 7.12 ± 0.02 to approximately 4.9, after which it stabilized. The most pronounced increase in TAC occurred during the first 72 h, peaking on the fourth day at 3.97 ± 0.12 g/kg before reaching a plateau. These findings indicate that *B. subtilis* 4-3 exhibited notable adaptability to the corn flour substrate, effectively metabolizing the available carbon sources to produce organic acids.

The accumulation of four functional components showed a gradual increase over the fermentation period (0–5 d), with the accumulation curve predominantly resembling an “S” shape. This indicates that the accumulation of functional components was slow during both the initial and final stages of fermentation, while experiencing rapid growth during the mid-stage. The accumulation rate of these functional components reflects, to a certain extent, the metabolic activity and growth status of the microorganisms. Therefore, the Gompertz model was applied to analyze the four functional components, with the results shown in Table 2. The table reveals that the Gompertz model provided a strong fit for the FPC, BPC, and TAC (*R*^2^ ≥ 0.92), a moderate fit for the TFC (*R*^2^ = 0.83), and the weakest fit for the TPC. According to the predictions of the Gompertz model, the maximum content of TAC and FPC can reach 4.07 g/kg and 32.74 mg/g, respectively, which closely align with the actual measured values of 3.99 ± 0.17 g/kg and 33.14 ± 1.84 mg/g. The maximum measured values for TFC and TPC were 7.56 ± 0.29 mg/g and 39.5 ± 1.36 mg/g, respectively, which deviate from the predicted values. This discrepancy may be due to the decrease in content during the later stages of fermentation (5–7 d).

### 3.2. Microstructural Changes in Cornmeal Fermented with B. subtilis

**Figure 2:**
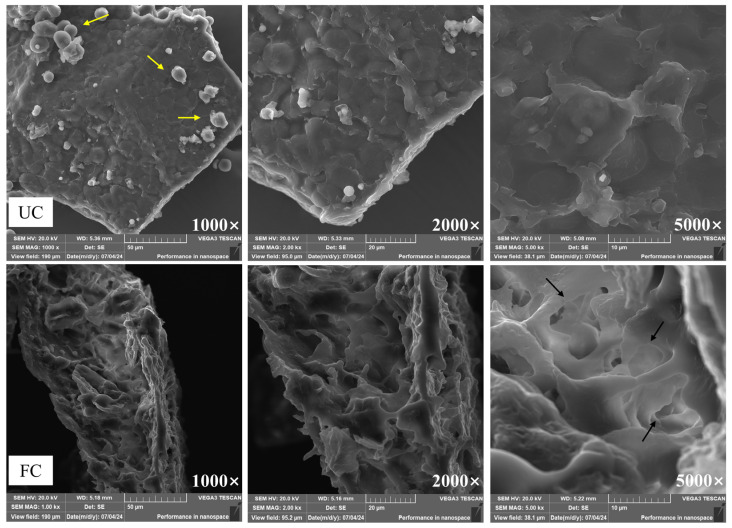
Microstructure of unfermented cornmeal and cornmeal fermented with *B. subtilis.* Note: The yellow arrow indicates starch granules, while the black arrow points to the honeycomb-like porous structure.

Cornmeal fermented with *B. subtilis* showed significant alterations in its microstructure (Figure 2). The unfermented cornmeal displayed a compact cellulosic wall with larger, block-like structures, characterized by a smooth surface and unevenly distributed spherical starch granules. In contrast, the surface of the FC appeared disintegrated and irregular, indicating a substantial disruption of the cellulosic wall structure. A loose, porous, honeycomb-like structure emerged, and the spherical starch granules were noticeably absent. These alterations suggest that fermentation by *B. subtilis* significantly disrupted the microstructural integrity of cornmeal, resulting not only in changes to its physical properties but also in a potential improvement of its nutritional quality and digestibility.

### 3.3. The Thermodynamic and Structural Analysis

**Figure 3:**
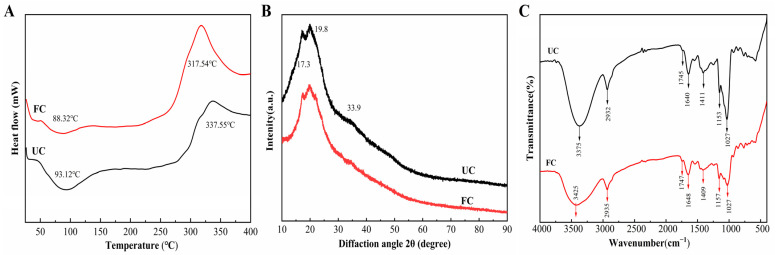
Thermodynamic and structural analysis of fermented cornmeal. (**A**) DSC, (**B**) XRD, (**C**) FT-IR of UC and FC.

The thermodynamic profiles of unfermented cornmeal and fermented cornmeal displayed significant differences (Figure 3A). Both samples exhibited an initial endothermic peak in the temperature range of 50–125 °C. The peak temperature for unfermented cornmeal was recorded at 93.12 °C, while the peak for fermented cornmeal, which had undergone fermentation with *B. subtilis* for 5 days, was observed at a lower temperature of 88.32 °C. Additionally, both unfermented cornmeal and fermented cornmeal demonstrated an exothermic peak within the 250–400 °C range, with unfermented cornmeal reaching a peak temperature of 337.55 °C, whereas fermented cornmeal exhibited a significantly lower peak temperature in this region.

The crystal structures and crystallinities of unfermented cornmeal and fermented cornmeal were analyzed using XRD (Figure 3B). The crystal structures of both samples exhibited remarkable similarity, characterized by diffraction peaking at 2*θ* = 17.3°, 19.8°, and 33.9° for both cornmeal types. Although fermentation did not alter the type of crystal structure in cornmeal, the diffraction peak intensities in fermented cornmeal were consistently weaker than those in unfermented cornmeal.

The FT-IR spectra of cornmeal before and after fermentation are presented in (Figure 3C). Both unfermented cornmeal and fermented cornmeal exhibited similar spectral profiles, with minor variations in the intensity and position of certain absorption peaks. A broad and intense absorption peak at 3375 cm^−1^ was observed, which corresponds to intermolecular hydrogen bonds, -OH groups associated with dietary fiber chains, and the stretching and bending vibrations of O-H in starch. This observation indicates the disruption of intermolecular hydrogen bonds in cellulose and hemicellulose present in dietary fibers. The absorption peak at 1745 cm^−1^ corresponds to the stretching vibrations of C=O in carboxylic acids or xylan esters. Furthermore, the characteristic absorption peak at 1640 cm^−1^ indicates the C=O in the benzene ring of lignin. Changes in this peak, such as shift, decreased in intensity, and alterations in area may suggest the degradation of ester bonds between polyphenols and lignin during fermentation. The peaks observed between 1411 cm^−1^ and 1260 cm^−1^ correspond to the weak intensity absorptions produced by the intramolecular bending vibrations of -OH within the cellulose chain. Finally, the peaks at 1153 cm^−1^ and 1027 cm^−1^ represent the elastic vibrations of C-C in polysaccharide molecules.

### 3.4. Dynamic Changes in Enzymatic Activities During Fermentation

**Figure 4:**
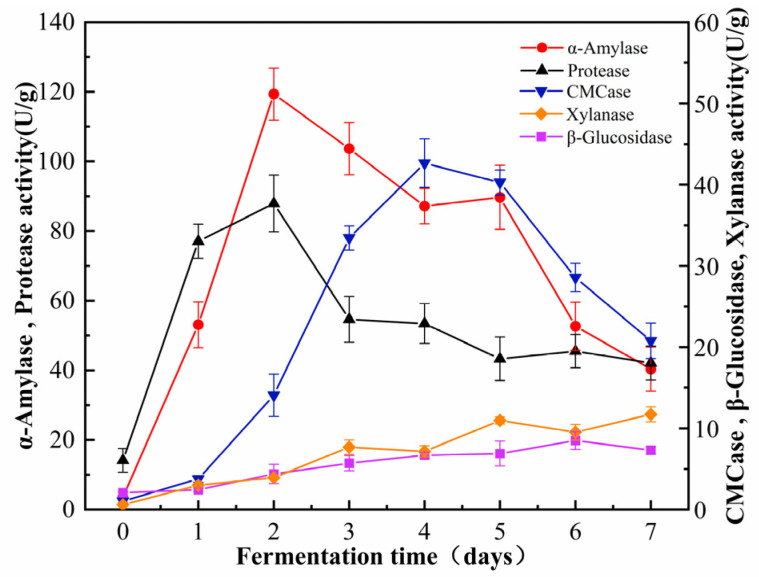
The dynamic changes in related enzyme activity during the fermentation process.

During the fermentation process of *B. subtilis*, distinct trends were observed in the activities of its five predominant hydrolytic enzymes (Figure 4). During the initial phase of fermentation, the activities of protease and amylase increased significantly, while the activities of cellulase, xylanase, and *β*-glucosidase increased at a comparatively slower rate. The delayed increase in these enzyme activities may indicate a prioritization of protein and starch degradation over the processing of cellulose and hemicellulose during the initial phase of fermentation. The activity of *α*-amylase was relatively low in the unfermented cornmeal, measuring 3.55 ± 1.48 U/g. However, fermentation significantly enhanced *α*-amylase activity, particularly after 48 h, when it peaked at 119.36 ± 7.50 U/g. Following this peak, a subsequent decline in activity was observed. Functionally, *α*-amylase randomly cleaves *α*-1,4-glycosidic bonds in starch, glycogen, and related polysaccharides, thereby yielding oligosaccharides of varying lengths. The dynamic changes in *α*-amylase activity throughout fermentation reflect the organism’s adaptability and efficiency in utilizing available resources.

During the liquid-state fermentation process of *B. subtilis*, both cellulase and *β*-glucosidase were produced in measurable quantities, with the activity of cellulase significantly surpassing that of *β*-glucosidase. Both hydrolytic enzymes exhibited a characteristic activity pattern, characterized by an initial increase followed by a subsequent decline. The activity of cellulase reached its maximum on the 4th day of fermentation, achieving a value of 42.65 ± 2.98 U/g. In contrast, the activity of *β*-glucosidase peaked on the 5th day, recording a value of 12.68 ± 1.61 U/g. Although this peak was significant, it was markedly lower than that of cellulose.

### 3.5. Changes in Antioxidant Capacity During Fermentation

**Figure 5:**
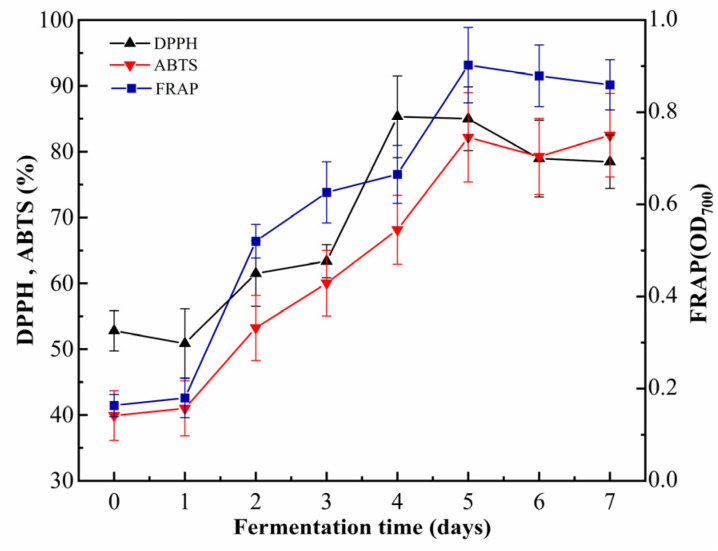
The dynamic changes in antioxidant activity during the fermentation. Note: DPPH, ABTS, and FRAP correspond to the measurements of DPPH radical scavenging ability, ABTS radical scavenging ability, and ferric reducing antioxidant power, respectively.

Fermentation significantly enhanced the antioxidant activity of cornmeal, as evidenced by various assays, including DPPH, ABTS^+^, and FRAP (Figure 5). Throughout the fermentation period from 0 to 4 days, the DPPH radical scavenging rate steadily increased from 52.78 ± 3.07% to 85.31 ± 6.21%. This rate stabilized from 4 to 7 d before experiencing a slight decline. Similarly, the ABTS^+^ radical scavenging rate increased from 39.92 ± 3.78% at day 0 to 82.19 ± 6.77% by day 5, indicating a robust enhancement. Concurrently, the FRAP value demonstrated significant improvement, increasing from 0.163 ± 0.02 to 0.90 ± 0.08, after which it stabilized. Notably, compared to the unfermented cornmeal, the ABTS^+^ radical scavenging rate of fermented cornmeal exhibited a remarkable increase of 42.27%, effectively doubling its original value. Furthermore, the reducing power measured by FRAP showed an enhancement of approximately 5.5 times, thereby illustrating the potent antioxidant capacity imparted through fermentation.

### 3.6. Correlation Analysis Between Functional Components, Hydrolase Activity, and Antioxidant Capacity of Fermented Cornmeal

**Figure 6:**
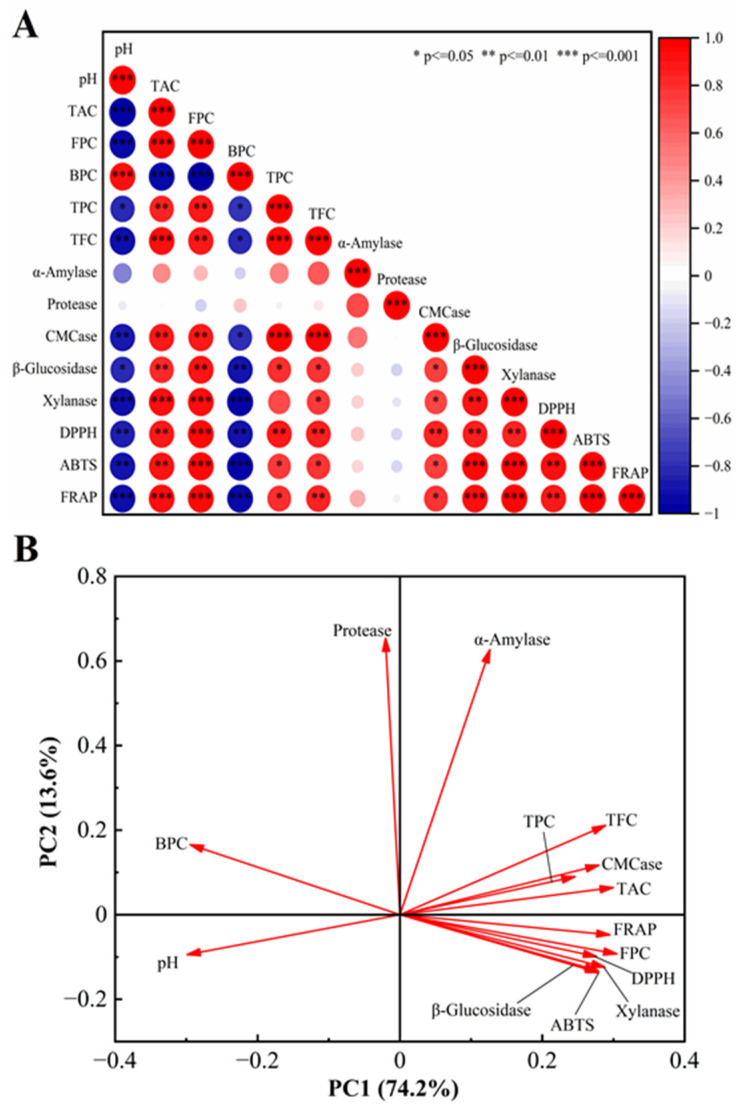
Pearson correlation analysis and PCA among functional components, hydrolase activity, and antioxidant activity. (**A**) Pearson correlation analysis between two variables. The size of the circle reflects the strength of the correlation between the variables. A larger circle corresponds to a stronger correlation, whereas a smaller circle signifies a weaker correlation. (**B**) the two-dimensional PCA plot.

Pearson correlation analysis and PCA were utilized to assess the relationships among functional components, hydrolytic enzyme activities, and antioxidant capacities in fermented cornmeal (Figure 6). Free phenols in fermented cornmeal demonstrated a strong positive correlation with the activities of *β*-glucosidase (*r* = 0.92, *p* < 0.01), xylanase (*r* = 0.94, *p* < 0.01), and cellulase (*r* = 0.90, *p* < 0.01), while bound phenols showed a significant negative correlation with these three enzymes. The correlation between *α*-amylase and soluble polyphenols was weak (*r* = 0.29, *p* > 0.05), whereas its correlation with TFC was moderate (*r* = 0.66, *p* < 0.05). Structural modifications of polyphenols, including hydroxylation, glycosylation, methylation, and methoxylation, influenced the activity of *α*-amylase. Furthermore, free phenols were highly positively correlated with antioxidant capacities. The contribution rate of free phenols in cornmeal increased from 20.24% to 83.98%, while the contribution rate of bound phenols decreased from 79.76% to 16.02%. These changes in contribution rates directly correlate with the observed variations in antioxidant activities, indicating that enhancing the free phenol content may enhance antioxidant properties in fermented cornmeal. Additionally, the total acidity content exhibited a significant positive correlation with antioxidant capacities.

The PCA plot revealed that principal components 1 and 2 (PC1 and PC2) accounted for a substantial portion of the contribution rate, specifically 74.2% and 13.6%, respectively, with a cumulative contribution of 87.8% (Figure 6B). This indicates that the PCA plot effectively captured the differences among the various analyzed components. TFC, TPC, TAC, FPC, and the three carbohydrate enzymes (*β*-glucosidase, xylanase, and CMCase) showed strong positive correlations with PC1, while BPC and pH exhibited negative correlations with PC1, suggesting that PC1 was primarily influenced by these components. Additionally, protease and *α*-amylase displayed strong positive correlations with PC2, indicating a significant correlation between PC2 and these two enzymes. Furthermore, the FPC was categorized with antioxidant indices, suggesting that the free phenol content in fermented cornmeal had the most substantial impact on its antioxidant activity. This analysis was consistent with the results of Pearson correlation analysis.

## 4. Discussion

Fermentation with *B. subtilis* significantly increased TPC and TFC in cornmeal, particularly enhancing FPC. This suggests that *B. subtilis* fermentation effectively converted insoluble bound phenols in corn into soluble free phenols [37]. Similar results were observed with oats fermented by *M. purpureus* and *B. subtilis*, which led to an increase in FPC and a decrease in levels of BPC [24]. It is well established that there is a distinction in solubility and bioavailability between bound and free phenols [38]. The increase in FPC enhances the bioavailability of food polyphenols, offering greater health benefits [39]. Therefore, the fermentation of cornmeal by *B. subtilis* represents a promising approach to improving its functional properties by increasing the bioavailable free phenols. This enhancement could potentially lead to greater health benefits from dietary polyphenols, thus adding value to fermented cornmeal products.

To deepen our understanding of the regulatory effects of *B. subtilis* on phenolic compounds in cornmeal, we employed SEM to examine both unfermented cornmeal and fermented cornmeal. Our findings suggested that *B. subtilis* fermentation disrupted the microsurface structure of cornmeal. It is well established that most phenolic compounds in corn are tightly bound to cell wall constituents through hydrogen bonds, ether bonds, and covalent bonds [40]. Additionally, the reduction in the initial endothermic peak temperature of fermented cornmeal was attributed to the evaporation of bound water within the samples [41]. This suggested that fermentation may have compromised the microstructure of the cornmeal, thereby diminishing its affinity for water molecules. Both unfermented cornmeal and fermented cornmeal displayed an exothermic peak within the 250–400 °C range, primarily caused by the pyrolysis of cellulose and hemicellulose [26,42]. Notably, the peak temperature in fermented cornmeal was considerably lower than that in unfermented cornmeal, indicating that fermentation reduced the thermal stability of the sample. This reduction in thermal stability may result from the microbial decomposition of polysaccharides, particularly the hemicellulose component, leading to the release of associated bioactive compounds [43]. Bound phenolic compounds, characteristic of cereals, are known to be covalently linked to dietary fiber through various chemical bonds, including hydrogen bonds, ester bonds, and ether bonds [44]. Although fermentation did not alter the type of crystal structure, the diffraction peak intensities in fermented cornmeal were consistently weaker than those in unfermented cornmeal, suggesting a reduction in the crystallinity of starch and dietary fiber within the cornmeal and a disruption of the ordered crystal structure during fermentation [45]. Furthermore, the established relationships between hydrogen bonds, ester bonds, ether bonds, and the composition of dietary fiber imply that the interactions between bound phenolic and polysaccharides may also be altered by fermentation. Both unfermented cornmeal and fermented cornmeal exhibited similar shapes in their FT-IR spectra with only slight variations in the intensity and position of certain absorption peaks. Therefore, the fermentation process by *B. subtilis* disrupted the chemical bonds between polysaccharide molecules, facilitating the release of bound polyphenols that were connected to starch, cellulose, hemicellulose, and lignin through hydrogen bonds, ester bonds, ether bonds, and intermolecular forces. Consequently, it can be inferred that changes in the crystal structure and crystallinity of starch and dietary fiber are likely related to the release of bound phenolic.

The alteration of enzymatic activities plays a pivotal role in the catalytic release of phenolic compounds from grains during fermentation [46]. Therefore, we monitored the activities of five predominant hydrolytic enzymes produced by *B. subtilis* throughout the fermentation process to investigate the impact of these enzymes on phenolic compound release. *α*-Amylase randomly cleaves the *α*-1,4-glycosidic bonds in starch molecules, facilitating their breakdown into dextrins, oligosaccharides, and monosaccharides [47]. This process disrupts the structure of starch granules, thereby releasing phenolic compounds that were originally encapsulated within them. Cellulase effectively degrades the fibrous network of corn by breaking *β*-1,4-glycosidic bonds, thus reducing cellulose to smaller cellobiose and glucose [48]. This process not only disrupts the binding of phenolic components with cellulose, thereby promoting the release of phenolic compounds [49], but also loosens the large molecular polysaccharide structure within the corn matrix, thereby facilitating additional enzymatic actions. Furthermore, the degradation of corn fiber increases the specific surface area available for solvent contact with active substances, thereby enhancing the permeation and extraction of internal materials [50]. Additionally, *β*-glucosidase hydrolyzes glycosidic bonds, particularly breaking down cellobiose and other small-molecular-weight fiber dextrins into glucose [51]. The disruption of glycosidic structures not only facilitates the release of small molecular sugars but also indirectly influences the release of bound phenolic compounds. During fermentation, the degradation of the corn fiber may liberate phenolic compounds that are bound to polysaccharides. The resulting oligosaccharides provide energy for microbes, thus promoting further enzymatic hydrolysis of phenolic constituents [52,53]. This indicates that the presence of cellulase, *β*-glucosidase, and xylanase may be key to converting insoluble bound phenols into soluble free phenols in cornmeal [24]. Remarkably, protease activity peaks after 48 h, aligning with previous studies that identified a peak in protease production by *B. subtilis* between 24 and 48 h of fermentation [54,55]. The breakdown of proteins into smaller peptides and amino acids effectively converts protein-bound polyphenols into free phenols, which may be one of the primary factors facilitating the release of phenolic compounds [56]. The trends in hydrolytic enzyme production during fermentation closely correlated with changes in the TPC and TFC of the products, further indicating the relationship between phenolic compound release and the hydrolytic enzyme system throughout fermentation. In the later stages of fermentation, the observed increase in xylanase activity may be attributed to the initially limited secretion of xylanase by *B. subtilis* for polyphenol release. However, as the substrates for other enzymes are gradually depleted and xylobiose accumulated, the activity of xylanase becomes more pronounced [57]. Xylanase effectively degrades xylan in the corn into oligosaccharides and xylose [58]. This degradation disrupts the cell wall structure, thereby releasing bioactive compounds bound to xylan [59]. Observations from scanning electron microscopy reveal that the microstructure of cornmeal transitions from a smooth texture to a honeycomb-like appearance, indicating structural breakdown of the cell wall. Thus, the changes in enzymatic activity during fermentation have a dramatic influence on the release of phenolic compounds, indicating the intricate interplay between enzyme production and phenolic bioavailability.

The antioxidant activity of cornmeal was significantly enhanced by fermentation with *B. subtilis*. This enhancement can be attributed primarily to TAC and pH levels. A lower pH not only improved safety by inhibiting pathogenic growth but also extended the product’s shelf life [40,48]. *B. subtilis* exhibited good adaptability to the cornmeal substrate, efficiently metabolizing available carbon sources to generate organic acids, such as acetic and butyric acid. TAC showed a highly significant positive correlation with DPPH and ABTS^+^ radical scavenging rates, as well as with FRAP. These organic acids possess distinct functional properties that contribute to antioxidant activity [60]. Furthermore, *B. subtilis* effectively generated organic acids during fermentation [61]. Another critical finding was that free phenols displayed a strong positive correlation with DPPH and ABTS^+^ radical scavenging activity and FRAP levels. This correlation resulted from the fermentation process converting insoluble polyphenols into free polyphenols. Consequently, the enhanced antioxidant capacity of fermented cornmeal can be attributed to the ability of *B. subtilis* to release the bound phenolic compounds in corn into a free state while converting a greater number of hydroxyl groups, significantly enhancing their antioxidant activity [62,63]. Similar enhancement in the antioxidant activity has been observed in other substrates, such as rose residue after fermentation with *B. subtilis* [64]. This indicates the importance of enzymes, including cellulase, *β*-glucosidase, and xylanase, which facilitate the conversion of insoluble bound phenols to soluble free phenols in cornmeal. Research has found that changes in oat phenolic components are closely correlated with the activities of various carbohydrate hydrolases during the co-fermentation of oats [24]. Furthermore, a significant portion of insoluble bound phenols in cereals is covalently bound to cell wall components such as polysaccharides, cellulose, pectin, and lignin, through ester bonds [7,65]. *B. subtilis* enhances the activity of cellulase, *β*-glucosidase, and xylanase during the fermentation of oat bran, facilitating the release of phenolic compounds and increasing the concentration of organic acids, thereby strengthening their antioxidant activity [65]. Therefore, the fermentation of cornmeal with *B. subtilis* significantly enhances its antioxidant properties through biochemical transformations that improve the availability of bioactive compounds.

## 5. Conclusions

In the fermentation of cornmeal with *B. subtilis*, there was a considerable rise in the content of soluble free phenols, accompanied by a notable decrease in the content of insoluble bound phenols. This change in phenolic substances was closely associated with the activities of key enzymes, such as cellulase, *β*-glucosidase, and xylanase, which are essential for mobilizing phenolic substances. Utilizing techniques such as SEM, DSC, XRD and FT-IR, it is hypothesized that the enzymes produced by microorganisms disrupt the interactions between bound phenols and the food matrix. This disruption facilitates the release of phenolic compounds, thereby increasing their bioavailability. Consequently, the antioxidant activity of fermented cornmeal was significantly enhanced. These findings contribute to the ongoing research and development of functional foods aimed at enhancing health benefits through improved phenolic content.

## Figures and Tables

**Table 1:** Content of basic nutritional and functional components in corn.

Classification	Components	Content
Basic nutritional components (g/100 g)	Moisture	8.53 ± 0.39
	Starch	67.21 ± 0.36
	Ash	1.34 ± 0.10
	Crude fiber	3.34 ± 0.17
	Crude fat	4.38 ± 0.04
	Crude protein	8.31 ± 0.44
	Soluble sugars	10.54 ± 1.06
	Reducing sugars	3.95 ± 0.2
Functional components	FPC (GAE mg/g)	5.06 ± 0.36
	BPC (GAE mg/g)	23.02 ± 0.85
	TPC (GAE mg/g)	28.08 ± 1.21
	TFC (RE mg/g)	1.86 ± 0.09
	TAC (g/kg)	0.17 ± 0.01

Note: The results are expressed as mean ± SD (*n* = 3). Moisture content was measured from naturally air-dried corn samples, while other components were measured by dry matter.

**Table 2:** Estimation of the Gompertz model parameters and goodness of fit for the accumulation curve of functional components.

Components	*A*	*B*	*K*	*R* ^2^
TPC	38.14	7.22	−4.87	0.61
TFC	6.78	2.21	−0.75	0.83
FPC	32.74	8.48	0.13	0.92
BPC	83.91	−4.34	6.24	0.92
TAC	4.07	1.39	0.18	0.98

Note: *A* represents the maximum accumulation amount, *B* is a constant scale factor, *K* denotes the absolute accumulation rate, and *R*^2^ indicates the goodness of fit.

## Data Availability

The original contributions presented in this study are included in the article. Further inquiries can be directed to the corresponding authors.

## Abbreviations

The following abbreviations are used in this manuscript:

SEM	Scanning electron microscopy
DSC	Differential scanning calorimetry
XRD	X-ray diffraction
FT-IR	Fourier-transform infrared spectroscopy
FC	Fermented cornmeal
UC	Unfermented cornmeal
TAC	Total acid content
FPC	Free phenolic content
BPC	Bound phenolic content
TPC	Total phenolic content
TFC	Total flavonoid content
DPPH	1,1-diphenyl2-picryl hydrazyl
FRAP	Ferric reducing antioxidant power

## References

[1] Yi L., Shenjiao Y., Shiqing L., Xinping C., Fang C. (2010). Growth and development of maize (*Zea mays* L.) in response to different field water management practices: Resource capture and use efficiency. Agric. For. Meteorol..

[2] Deepak T.S., Jayadeep P.A. (2022). Prospects of Maize (Corn) Wet milling by-products as a source of functional food ingredients and nutraceuticals. Food Technol. Biotechnol..

[3] Erenstein O., Jaleta M., Sonder K., Mottaleb K., Prasanna B.M. (2022). Global maize production, consumption and trade: Trends and R&D implications. Food Secur..

[4] Siyuan S., Tong L., Liu R. (2018). Corn phytochemicals and their health benefits. Food Sci. Hum. Wellness.

[5] Hu Q.P., Xu J.G. (2011). Profiles of carotenoids, anthocyanins, phenolics, and antioxidant activity of selected color waxy corn grains during maturation. J. Agric. Food Chem..

[6] Acosta-Estrada B.A., Gutiérrez-Uribe J.A., Serna-Saldívar S.O. (2014). Bound phenolics in foods, a review. Food Chem..

[7] Wang Z., Li S., Ge S., Lin S. (2020). Review of distribution, extraction methods, and health benefits of bound phenolics in food plants. J. Agric. Food. Chem..

[8] Ed Nignpense B., Francis N., Blanchard C., Santhakumar A.B. (2021). Bioaccessibility and bioactivity of cereal polyphenols: A review. Foods.

[9] Shahidi F., Yeo J.D. (2016). Insoluble-bound phenolics in food. Molecules.

[10] Gironi F., Piemonte V. (2011). Temperature and solvent effects on polyphenol extraction process from chestnut tree wood. Chem. Eng. Res. Des..

[11] Yang F., Chen C., Ni D., Yang Y., Tian J., Li Y., Chen S., Ye X., Wang L. (2023). Effects of fermentation on bioactivity and the composition of polyphenols contained in polyphenol-rich foods: A review. Foods.

[12] Liu L., Zhang R., Deng Y., Zhang Y., Xiao J., Huang F., Wen W., Zhang M. (2017). Fermentation and complex enzyme hydrolysis enhance total phenolics and antioxidant activity of aqueous solution from rice bran pretreated by steaming with alpha-amylase. Food Chem..

[13] Lin S., Zhang X., Wang J., Li T., Wang L. (2024). Effect of lactic acid bacteria fermentation on bioactive components of black rice bran (*Oryza sativa* L.) with different milling fractions. Food Biosci..

[14] Wang L., Bei Q., Wu Y., Liao W., Wu Z. (2017). Characterization of soluble and insoluble-bound polyphenols from *Psidium guajava* L. leaves co-fermented with *Monascus anka* and *Bacillus sp*. and their bio-activities. J. Funct. Foods.

[15] Xiao L., Yang C., Zhang X., Wang Y., Li Z., Chen Y., Liu Z., Zhu M., Xiao Y. (2023). Effects of solid-state fermentation with *Bacillus subtilis* LK-1 on the volatile profile, catechins composition and antioxidant activity of dark teas. Food Chem. X.

[16] Juan M.-Y., Chou C.-C. (2010). Enhancement of antioxidant activity, total phenolic and flavonoid content of black soybeans by solid state fermentation with *Bacillus subtilis* BCRC 14715. Food Microbiol..

[17] (2021). National Food Safety Standard—Determination of Total Acid in Foods.

[18] Bei Q., Liu Y., Wang L., Chen G., Wu Z. (2017). Improving free, conjugated, and bound phenolic fractions in fermented oats (*Avena sativa* L.) with Monascus anka and their antioxidant activity. J. Funct. Foods.

[19] Li W.X., Zhang M.W., Jia X.C., Zhang M., Chen Y.X., Dong L.H., Huang F., Ma Q., Zhao D., Zhang R.F. (2024). Free and bound phenolic profiles of *Radix Puerariae Thomsonii* from different growing regions and their bioactivities. Food Chem. X.

[20] Akbari M., Razavi S.H., Khodaiyan F., Blesa J., Esteve M.J. (2023). Fermented corn bran: A by-product with improved total phenolic content and antioxidant activity. LWT-Food Sci. Technol..

[21] Liu X., Liu Y., Shan C., Yang X., Zhang Q., Xu N., Xu L., Song W. (2022). Effects of five extraction methods on total content, composition, and stability of flavonoids in jujube. Food Chem. X.

[22] Bei Q., Chen G., Lu F., Wu S., Wu Z. (2018). Enzymatic action mechanism of phenolic mobilization in oats (*Avena sativa* L.) during solid-state fermentation with *Monascus anka*. Food Chem..

[23] Xie J., Liu S., Dong R., Xie J., Chen Y., Peng G., Liao W., Xue P., Feng L., Yu Q. (2021). Bound polyphenols from insoluble dietary fiber of defatted rice bran by solid-state fermentation with trichoderma viride: Profile, activity, and release mechanism. J. Agric. Food. Chem..

[24] Chen G., Liu Y., Zeng J., Tian X., Bei Q., Wu Z. (2020). Enhancing three phenolic fractions of oats (*Avena sativa* L.) and their antioxidant activities by solid-state fermentation with *Monascus anka* and *Bacillus subtilis*. J. Cereal Sci..

[25] Qin B.S., He H.Y., Li Y.T., Fu Y., Qin Y.L. (2024). Screening and identification of β-glucosidase-producing strain and enzymatic properties of crude enzyme. China Brew..

[26] Wen Y., Niu M., Zhang B., Zhao S., Xiong S. (2017). Structural characteristics and functional properties of rice bran dietary fiber modified by enzymatic and enzyme-micronization treatments. LWT Food Sci. Technol..

[27] Xie M., Jie C., Shi T., Zhang L., Yu M. (2024). Effects of combined fermentation and ultrasonic treatments on the structure and properties of okara soluble dietary fiber. Food Chem. Adv..

[28] Peng G., Gan J., Dong R., Chen Y., Xie J., Huang Z., Gu Y., Huang D., Yu Q. (2021). Combined microwave and enzymatic treatment improve the release of insoluble bound phenolic compounds from the grapefruit peel insoluble dietary fiber. LWT-Food Sci. Technol..

[29] Ajila C.M., Prasada Rao U.J.S. (2013). Mango peel dietary fibre: Composition and associated bound phenolics. J. Funct. Foods.

[30] Zheng Z., Wei L., Zhu M., Qian Z., Liu J., Zhang L., Xu Y. (2023). Effect of lactic acid bacteria co-fermentation on antioxidant activity and metabolomic profiles of a juice made from wolfberry and longan. Food Res. Int..

[31] Ni D., Chen C., Yang Y., Tian J., Tu H., Yang F., Ye X. (2024). Changes in polyphenols and antioxidant activity in fermentation substrate during maotai-flavored liquor processing. Foods.

[32] Tan Y.M., Gao M.X., Li L., Jiang H.B., Liu Y.B., Gu T., Zhang J.L. (2024). Functional components and antioxidant activity were improved in ginger fermented by *Bifidobacterium adolescentis* and *Monascus purpureus*. LWT Food Sci. Technol..

[33] Wang L., Zhang J., Zhang W., Lin X., Li C., Wu Z. (2019). Role of carbohydrate-cleaving enzymes in phenolic mobilization of guava leaves tea during solid state bio-processing with *Monascus anka* and *Bacillus* sp. Process Biochem..

[34] Kasperek M.C., Velasquez Galeas A., Caetano-Silva M.E., Xie Z., Ulanov A., La Frano M., Devkota S., Miller M.J., Allen J.M. (2024). Microbial aromatic amino acid metabolism is modifiable in fermented food matrices to promote bioactivity. Food Chem..

[35] Kallscheuer N., Vogt M., Marienhagen J. (2017). A novel synthetic pathway enables microbial production of polyphenols independent from the endogenous aromatic amino acid metabolism. ACS Synth. Biol..

[36] Dueñas M., Surco-Laos F., González-Manzano S., González-Paramás A.M., Gómez-Orte E., Cabello J., Santos-Buelga C. (2013). Deglycosylation is a key step in biotransformation and lifespan effects of quercetin-3-O-glucoside in *Caenorhabditis elegans*. Pharmacol. Res..

[37] Chen G., Chen B., Song D. (2021). Co-microbiological regulation of phenolic release through solid-state fermentation of corn kernels (*Zea mays* L.) to improve their antioxidant activity. LWT-Food Sci. Technol..

[38] Wang T., He F., Chen G. (2014). Improving bioaccessibility and bioavailability of phenolic compounds in cereal grains through processing technologies: A concise review. J. Funct. Foods.

[39] Chen L., Guo Y., Li X., Gong K., Liu K. (2021). Phenolics and related in vitro functional activities of different varieties of fresh waxy corn: A whole grain. BMC Chem..

[40] Van Hung P. (2016). Phenolic compounds of cereals and their antioxidant capacity. Crit. Rev. Food Sci. Nutr..

[41] Zhang W., Zeng G., Pan Y., Chen W., Huang W., Chen H., Li Y. (2017). Properties of soluble dietary fiber-polysaccharide from papaya peel obtained through alkaline or ultrasound-assisted alkaline extraction. Carbohydr. Polym..

[42] Fan X., Chang H., Lin Y., Zhao X., Zhang A., Li S., Feng Z., Chen X. (2020). Effects of ultrasound-assisted enzyme hydrolysis on the microstructure and physicochemical properties of okara fibers. Ultrason. Sonochem..

[43] Si J., Xie J., Zheng B., Xie J., Chen Y., Yang C., Sun N., Wang Y., Hu X., Yu Q. (2023). Release characteristic of bound polyphenols from tea residues insoluble dietary fiber by mixed solid-state fermentation with cellulose degrading strains CZ-6 and CZ-7. Food Res. Int..

[44] Sejbuk M., Mironczuk-Chodakowska I., Karav S., Witkowska A.M. (2024). Dietary polyphenols, food processing and gut microbiome: Recent findings on bioavailability, bioactivity, and gut microbiome interplay. Antioxidants.

[45] Huang N., Ruan L., Zhang J., Wang Y., Shen Q., Deng Y., Liu Y. (2024). Improved physicochemical and functional properties of dietary fiber from matcha fermented by *Trichoderma viride*. Food Chem..

[46] Saharan P., Sadh P.K., Singh Duhan J. (2017). Comparative assessment of effect of fermentation on phenolics, flavanoids and free radical scavenging activity of commonly used cereals. Biocatal. Agric. Biotechnol..

[47] Yang Y., Jiao A., Zhao S., Liu Q., Fu X., Jin Z. (2021). Effect of removal of endogenous non-starch components on the structural, physicochemical properties, and in vitro digestibility of highland barley starch. Food Hydrocoll..

[48] Hu J., Tian D., Renneckar S., Saddler J.N. (2018). Enzyme mediated nanofibrillation of cellulose by the synergistic actions of an endoglucanase, lytic polysaccharide monooxygenase (LPMO) and xylanase. Sci. Rep..

[49] Bao Q., Yan J., Ma S. (2023). Effect of heat treatment on conformation and aggregation properties of wheat bran dietary fiber-gluten protein. Int. J. Biol. Macromol..

[50] Chen X., Tang W., Li X., Zhuang K., Lyu Q., Ding W. (2023). Effect of extrusion on phenolics from Jizi439 black wheat bran: The profile, structure, and bioactivities. LWT Food Sci. Technol..

[51] Capetti C.C.M., Vacilotto M.M., Dabul A.N.G., Sepulchro A.G.V., Pellegrini V.O.A., Polikarpov I. (2021). Recent advances in the enzymatic production and applications of xylooligosaccharides. World J. Microb. Biot..

[52] Wang Y., Wang Y., Zhou X., Du B., Chen Y. (2024). Release of bound phenol from *Rosa roxburghii* pomace via solid-state fermentation with *Trichoderma viride*: Mechanisms of change. Food Biosci..

[53] Li W., Zhang M., Zhang R., Huang F., Dong L., Jia X., Zhang M. (2024). Structural elucidation, binding sites exploration and biological activities of bound phenolics from *Radix Puerariae Thomsonii*. Food Chem..

[54] Reddy N., Deekonda V., Seshagiri S., Reddy R., Gangula A.K. (2022). Production, characterization and applications of proteases produced by *Bacillus licheniformis*, *Acinetobacter pittii* and *Aspergillus niger* using neem seed oil cake as the substrate. Ind. Crops Prod..

[55] Prakasham R.S., Rao C.S., Sarma P.N. (2006). Green gram husk—An inexpensive substrate for alkaline protease production by *Bacillus* sp. in solid-state fermentation. Bioresour. Technol..

[56] Shi L., Pico J., Zamani S., Castellarin S.D., Dee D.R. (2024). Fibrillization of lentil proteins is impacted by the protein extraction conditions and co-extracted phenolics. Food Chem..

[57] Nemes S.A., Mitrea L., Teleky B.-E., Dulf E.H., Călinoiu L.F., Ranga F., Elekes D.-G.-A., Diaconeasa Z., Dulf F.V., Vodnar D.C. (2025). Integration of ultrasound and microwave pretreatments with solid-state fermentation enhances the release of sugars, organic acids, and phenolic compounds in wheat bran. Food Chem..

[58] Martins M., Sganzerla W.G., Forster-Carneiro T., Goldbeck R. (2023). Recent advances in xylo-oligosaccharides production and applications: A comprehensive review and bibliometric analysis. Biocatal. Agric. Biotechnol..

[59] Kaur G., Kaur P., Kaur J., Singla D., Taggar M.S. (2024). Xylanase, xylooligosaccharide and xylitol production from lignocellulosic biomass: Exploring biovalorization of xylan from a sustainable biorefinery perspective. Ind. Crops Prod..

[60] Quiroga P.R., Nepote V., Baumgartner M.T. (2019). Contribution of organic acids to α-terpinene antioxidant activity. Food Chem..

[61] Gao T., Wong Y., Ng C., Ho K. (2012). L-lactic acid production by *Bacillus subtilis* MUR1. Bioresour. Technol..

[62] Bei Q., Wu Z., Chen G. (2020). Dynamic changes in the phenolic composition and antioxidant activity of oats during simultaneous hydrolysis and fermentation. Food Chem..

[63] Bian X., Xie X., Cai J., Zhao Y., Miao W., Chen X., Xiao Y., Li N., Wu J.-L. (2022). Dynamic changes of phenolic acids and antioxidant activity of Citri Reticulatae Pericarpium during aging processes. Food Chem..

[64] Hu Y., Wang X., Qin C., Li T., Liu W., Ren D. (2022). Fermentation of rose residue by *Lactiplantibacillus plantarum* B7 and *Bacillus subtilis* natto promotes polyphenol content and beneficial bioactivity. J. Biosci. Bioeng..

[65] Alrahmany R., Avis T.J., Tsopmo A. (2013). Treatment of oat bran with carbohydrases increases soluble phenolic acid content and influences antioxidant and antimicrobial activities. Food Res. Int..

